# Serologic indices of hepatitis B virus infection in military recruits in Greece (2004–2005)

**DOI:** 10.1186/1471-2334-6-163

**Published:** 2006-11-14

**Authors:** Vasilios German, Georgios Giannakos, Petros Kopterides, Konstantinos Liaskonis, Matthew E Falagas

**Affiliations:** 1 Department of Internal Medicine and Infectious Diseases, 401 Army General Hospital of Athens, Greece; 2 Alfa Institute of Biomedical Sciences (AIBS), Athens, Greece; 3 Department of Microbiology, 401 Army General Hospital of Athens, Greece; 4 Department of Medicine, Tufts University School of Medicine, Boston, Massachusetts, USA

## Abstract

**Background:**

The prevalence of hepatitis B virus infection in Greece has been decreasing over the last decades. However, recent epidemiological data are lacking.

**Methods:**

We studied 1,840 Army recruits from 05/2004 until 10/2005, and performed serological testing for HBsAg, anti-HBsAg, and anti-HBcAg. We also examined their association with several factors, including age, residential area, socioeconomic class, and educational level.

**Results:**

Mean age (± SD) of the recruits was 20.5 (± 2.1) years. Antibodies to HBV core antigen [anti-HBcAg (+)] were found in 31 (1.68%) of 1,840 participants. Only 6 (0.32%) were HBsAg (+)/anti-HBsAg (-)/anti-HBcAg (+), while 21 (1.14%) were HBsAg (-)/anti-HBsAg (+)/anti-HBcAg (+), and 4 (0.22%) were HBsAg (-)/anti-HBsAg (-)/anti-HBcAg (+). Overall, 1,144 recruits (62.17%) had antibodies against HBsAg [HBsAg (-)/anti-HBsAg (+)/anti-HBcAg (-)]; 665 recruits (36.14%) had undetectable anti-HBsAg levels. Multivariable analysis showed that younger age (OR: 0.87; 95% CI: 0.82–0.92) and advanced educational level (OR: 1.59; 95% CI: 1.32–1.93) were independently associated with serologic evidence suggestive of previous HBV vaccination.

**Conclusion:**

We document a further decline of the prevalence of chronic HBV infection among Greek military recruits, a fact that may support the effectiveness of the ongoing immunization programme.

## Background

Hepatitis B is a well-recognized global public health problem. It is estimated that nearly 2 billion people around the world have serologic evidence of past or present hepatitis B virus (HBV) infection, while 350 million people are chronically infected [1]. Ongoing efforts towards the decrease of the prevalence of HBV infection have led to implementation of vaccination in childhood. Given the fact that HBV is the main etiologic factor for hepatocellular carcinoma, epidemiological studies are needed to document the compliance to and effectiveness of HBV vaccination programs in various populations and settings in order to establish a cost-effective health policy.

Approximately thirty years ago, Greece had an intermediate prevalence of chronic HBV infection, as seropositivity for HBV surface antigen (HBsAg) was in the range of 2% to 7% [2-5]. However, epidemiological studies in the previous decade showed a decline of this prevalence to the levels of low endemicity – about 0.9% – partially attributed to changes of the socioeconomic conditions [3,6]. In addition, HBV vaccination programs were gradually implemented. Most private paediatricians, who administer more than 60% of all childhood vaccinations in Greece, began vaccinating children against HBV in their practices in 1993. Therefore, HBV vaccination had already been administered to 36% and 56% of children (aged 5–6 years' old) entering primary school in 1995 and 1997, respectively [7]. The government decided to launch a national HBV immunization programme and raise the financial burden of its implementation in 1998 [7]. At the same time, efforts were made to offer HBV vaccination to high-school students that apparently had not been immunized against HBV earlier in their life. Nowadays, vaccination has been considered as mandatory for all newborns and children entering puberty that were not immunized earlier in their life (about 12 years' old) [7].

In order to provide further information on current HBV epidemiology in Greece, we conducted a prospective study of young military recruits joining the Hellenic Army from May 2004 to October 2005. We performed serological testing for HBV infection markers and searched for their possible association with several epidemiological characteristics.

## Methods

### Study population

The study took place in Sparta, the historic capital city of ancient Spartans, where the Supply and Transportation Corps Training Centre of the Hellenic Army is currently based. The study population consisted of 1,840 men, aged 17–34 years, who were transferred to Sparta in order to receive training in food handling and meal preparation. The study protocol was approved by the Medical Directorate of the Hellenic Army General Staff.

### Laboratory testing

A blood sample was taken from all recruits, as part of their standard evaluation procedure in order to obtain a health certificate. Blood sampling was performed from May 2004 to October 2005, on the occasion of recruit enlistment and just for the purpose of this study. Ten milliliters of venous blood were collected in a dry test tube from each soldier. Blood samples were transported to the Microbiology Laboratory of 401 Army General Hospital of Athens, within 3 hours, for processing.

Sera were separated by centrifugation and kept frozen at -20°C, until tested. All sera were tested for hepatitis B surface antibody (anti-HBsAg) by a Microparticle Enzyme Immunoassay (MEIA) (AxSym AUSAB, Abbott Laboratories Diagnosis Division, Abbott Park, IL, USA), hepatitis B surface antigen (HBsAg), and hepatitis B core antibody (anti-HBcAg) by MEIA (AxSym HBsAg (V2) and AxSym CORE, Abbott Laboratories Diagnosis Division, Abbott Park, IL, USA, respectively), according to the manufacturer's instructions. All positive samples for HBsAg were analyzed twice with the same test. A positive test for antibodies to hepatitis B core antigen was considered to be merely suggestive of a history of HBV infection, while chronic HBV infection was defined by the simultaneous presence of both HBsAg and antibodies to hepatitis B core antigen. An anti-HBsAg titer of greater than 10 mIU/ml was considered protective [8,9]. All study participants were informed on test results by means of personal interviews, while infected patients were examined and appropriately counseled by the staff of the Internal Medicine and Infectious Diseases Department of 401 Army General Hospital of Athens.

### Data collection

Epidemiological data regarding age, geographic area of residence, educational level and prior occupation were collected by means of personal interviews. For the purposes of this analysis the residential areas were divided in urban (including the seven major cities of Greece, namely Athens, Thessaloniki, Patra, Iraklion, Larisa, Ioannina and Alexandroupolis) and rural (including all other smaller cities, towns and villages). The educational level stratifications we used were: elementary school, high school, vocational education institute, technical educational institute, and university.

The socioeconomic level was determined on the basis of the occupation prior to recruitment to the Army using the Erikson-Goldthorpe Portocarero (EGP) social class scheme [10]. According to this scheme, occupations are grouped in seven classes, with class I including higher-grade professionals, class II lower-grade professionals administrators, class III routine non-manual employees, class IV small proprietors, artisans, farmers and smallholders, class V lower-grade technicians, class VI manual workers, and class VII including unskilled manual workers and agricultural workers. We performed a slight modification of the EGP social class scheme, by adding one more class in order to include persons unemployed at the time of military recruitment. The recruits' vaccination history was unknown, as no medical records were available for review. Finally, demographical data were available for a subset of the participants.

### Statistical analysis

Chi-square test was used in the bivariable analysis for the comparison of the prevalence of HBV serologic markers in various subgroups based on discrete characteristics. Normality was assessed and t-test or a non-parametric test was used for normally and non-normally distributed continuous variables. A *p*-value <0.05 was considered statistically significant. A backward multivariable logistic regression model was used in order to estimate relationships between recruits' epidemiological factors and HBV infection markers. Data were analyzed using SPSS for Windows version 12.0 (SPSS Inc, Chicago, IL, USA).

## Results

All recruits were male, and their mean age (± SD) was 20.5 (± 2.1) years. Of the 1,840 study participants, 1,144 (62.17%) had antibodies against HBsAg [HBsAg (-)/anti-HBsAg (+)/anti-HBcAg (-)], while 665 recruits (36.14%) had undetectable anti-HBsAg levels. It should be noted that testing of the HBV serologic status is recommended in selected Greek army training centers and, notwithstanding the limitations of anti-HBsAg as a marker for vaccination status, the recruits found to have undetectable titers of anti-HbsAg are advised to receive HBV vaccination on a personal basis after consultation with a primary physician.

Antibodies to HBV core antigen were found in 31 recruits, or 1.68% of the total population under study (1,840 participants). Twenty-one of them (1.14% of the total population) were HBsAg (-)/anti-HBsAg (+)/anti-HBcAg (+), and 4 (0.22%) were HBsAg (-)/anti-HBsAg (-)/anti-HBcAg (+). Only 4 recruits were anti-HBs (-)/HBsAg (-). The overall prevalence of HbsAg carriage was 0.32%, or, 6 out of 1,840 participants. The results of the above-mentioned serologic analysis are shown in Table 1.

**Table 1:** Hepatitis B virus (HBV) infection serologic markers in 1,840 Greek army recruits (2004–2005).

HBV markers	N	% of the study population
anti-HBcAg (+)	31	1.68
HBsAg (+)/anti-HBsAg (-)	6	0.32
anti-HBsAg (+)/HBsAg (-)	21	1.14
anti-HBsAg (-)/HbsAg (-)	4	0.22
anti-HBsAg (+)/anti-HBcAg (-)/HBsAg (-)	1,144	62.17
anti-HBsAg (-)/anti-HBcAg (-)/HBsAg (-)	665	36.14

Analysis of data from 6 recruits who tested positive for HBsAg showed that 3 of them were 19 years old, 1 was 21 years old and 2 were 22 years old. Interestingly, 5 out of 6 were from rural Greece, and 2 were unemployed, while the other 4 belonged to classes VI and VII of the EGP social class scheme. No formal statistical analysis of these data was performed due to the small number of persons with HbsAg seropositivity.

The bivariable analysis we performed to investigate possible relationship of the available epidemiological characteristics with the presence of anti-HBcAg seropositivity yielded no statistically significant associations (*p *= 0.60 for socioeconomic class, *p *= 0.24 for residential area, *p *= 0.77 for educational level, and *p *= 0.29 for age).

**Figure 1:**
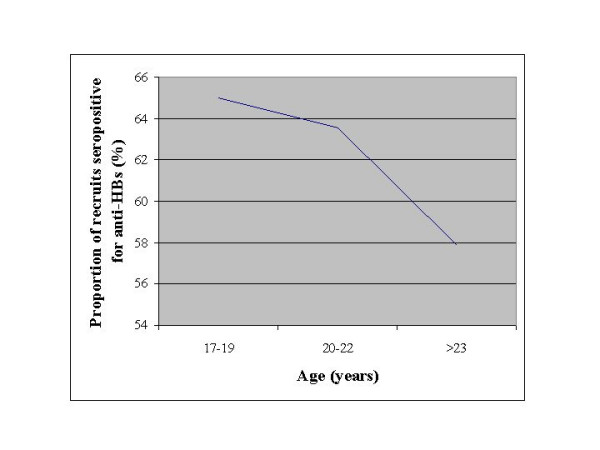
Prevalence of anti-HbsAg (+) in correlation with recruits' age.

A backward multivariable logistic regression model showed that younger age (OR: 0.87; 95% CI: 0.82–0.92) and advanced educational level (OR: 1.59; 95% CI: 1.32–1.93) were independently associated with anti-HbsAg seropositivity [anti-HBs (+)/anti-HBcAg (-)/HBsAg (-)]. We display this inverse correlation of anti-HBsAg seropositivity with age in Figure 1. No association was found with the area of residence and the socioeconomic class.

## Discussion

The main finding of our study is that compared with previous epidemiological data regarding HBV infection in Army recruits in Greece, we documented a further reduction of the prevalence of seropositivity for HBsAg during the last decade. In 1971, the HBsAg seroprevalence among 6,708 Hellenic Air Force recruits, tested consecutively and without any selection, was 4.9% [5]. In 1998, the overall prevalence of HBsAg positivity in 1,050 male navy recruits was 0.95% [3]. Our findings demonstrate a continuing trend in seroepidemiology of HBV in Greece, about 10 years after the establishment of mandatory vaccination for infants and adolescents not previously immunized. The results of our study are in accordance with a very recently published study in 13,581 Greek women at reproductive age (16–45 years old) in which HBsAg seroprevalence of 0.29% was reported [6].

It is noteworthy that the frequency of anti-HBsAg seropositivity was more common (62.2%) among our study subjects compared to the ones reported from previous studies in Greece. Serologic indices denoting previous vaccination were found in 32.6% of navy recruits in a study performed in 1998 [3], while only 12.2% of a general population (over 18 years old) studied in south-western Greece had such indices in 2002 [4]. The higher degree of anti-HBsAg seropositivity observed in our study may be a reflection of the success of the ongoing vaccination program in young people in Greece. However, measures should be taken in order to achieve universal vaccination.

Another interesting finding of our study is that younger age and higher educational level were independently associated with anti-HBsAg seropositivity. Even though the unavailability of the vaccination records does not allow us to refute the possibility that younger and more educated subjects were vaccinated at a time closer to their recruitment into the military, we believe that another explanation of this finding may be that the integration of HBV vaccination into routine vaccination schedules rendered results mainly in younger adolescents. Even though it is likely that the higher probability of vaccination is not actually related to the level of education of the recruits but rather to the higher educational level of their parents (i.e. more educated parents are more likely to vaccinate their children), the association between a higher educational level and vaccination rates observed in our study seems to be strong and it has also been noted in studies of other populations and for other types of vaccines. Our data suggest that stronger efforts should be made for implementing vaccination policies in unvaccinated adults, especially those with a low educational level.

Our study has several limitations. First, it should be noted that each study has its own "target" population, setting and methodology. For example, our analysis is focused at a population with specific characteristics, namely young male military recruits. Thus, are findings cannot be directly comparable with previous studies nor extrapolated to women or to special populations at high risk for HBV infection, such as intravenous drug users, sex workers, as well as hemodialysis patients and multiply transfused patients. In addition, our analysis did not include immigrants who do not have military obligations; some of them come from countries with a high prevalence of HBV. A high prevalence of HBV infection in refugees has been reported in previous studies [11,12]. There is a concern that, unless targeted vaccination programs for these communities are implemented, there will be an increase in the HBV incidence in the general population in the future. Also, we did not determine the viral load of the six HBsAg (+) recruits and we did not perform S-region sequencing in order to identify possible escape mutants. Another limitation of our findings is that the recruits' vaccination records were not available for further analysis, due to the special circumstances under which this study was conducted (limited time-frame in a military unit). This makes difficult the interpretation of the HBV serologic profile in some of the recruits. For example, it is known that the levels of anti-HbsAg decrease over time in vaccinated persons without this decline being associated with a loss of protection against HBV [13]. It is possible that some recruits with undetectable levels of anti-HBsAg were in fact vaccinated and, therefore, protected against HBV infection. Finally, we considered the presence of antibodies to hepatitis B core antigen in 31 recruits as an indication of previous HBV infection. This seems to be true for the 27 out of the 31 recruits that had other markers of HBV infection as well [6 with HBsAg (+) and 21 with anti-HBsAg (+)]. However, the 4 recruits with the "anti-HbcAg (+) alone" profile may fall under one of the following possibilities: (i) false positive cases, (ii) the "window phase" of an acute HBV infection, (iii) an HBV infection that resolved years or decades earlier, (iv) an unresolved chronic HBV infection with low grade, possibly intermittent virus production and detectable serum or liver HBV-DNA, and (v) suppression of HBV replication by a simultaneous HCV co-infection [14].

Despite the above limitations, the results of this study may reflect the continuing progress towards the eradication of HBV transmission in Greece through universal immunization. In recent years, the implementation of a vaccination program against HBV, medical precautions, screening of blood donors, and modifications in socioeconomic conditions, resulted in significant decline in HBV infection in Greece. Nevertheless, demographic changes resulting from the entrance in Greece of immigrants and repatriated people, mainly from countries of the Eastern Europe, as well as refugees from various countries with high endemicity for HBV infection, in the last decade, may lead to alterations of the epidemiology of HBV infection in our country. Therefore, the continuing surveillance of trends in the epidemiology of this inflection is of paramount importance for the design of effective vaccination policies in the future.

## Conclusion

Our data suggest that that the National Immunization Program for hepatitis B has been largely successful in reducing the prevalence of hepatitis B infection and increasing immunity levels in the population. However, hepatitis B virus infection still remains a serious public health problem. Therefore, there is a need to strengthen the hepatitis B immunization program to reach universal vaccination. The goal of eliminating HBV transmission is attainable and efforts towards this direction should be a top public health priority.

## Competing interests

The author(s) declare that they have no competing interests.

## Authors' contributions

VG, PK, and MEF had the idea for the study. VG and GG collected the relevant data. KL did the microbiological studies. MEF did the statistical analysis. VG, PK, and MEF wrote different parts of the first version of the manuscript. All authors made revisions of the manuscript and approved its final version.

## Pre-publication history

The pre-publication history for this paper can be accessed here:



## References

[B1] Alter MJ (2003). Epidemiology of hepatitis B in Europe and worldwide. J Hepatol.

[B2] Kyriakis KP, Foudoulaki LE, Papoulia EI, Sofroniadou KE (2000). Seroprevalence of hepatitis B surface antigen (HBsAg) among first-time and sporadic blood donors in Greece: 1991–1996. Transfus Med.

[B3] Stamouli M, Gizaris V, Totos G, Papaevangelou G (1999). Decline of hepatitis B infection in Greece. Eur J Epidemiol.

[B4] Gogos CA, Fouka KP, Nikiforidis G, Avgeridis K, Sakellaropoulos G, Bassaris H, Maniatis A, Skoutelis A (2003). Prevalence of hepatitis B and C virus infection in the general population and selected groups in South-Western Greece. Eur J Epidemiol.

[B5] Vissoulis H, Hadziyannis S, Papaevangelou G, Afroudakis A, Giannopoulos C, Gioustosi A, Merikas GL (1972). Geographical distribution of hepatitis-associated antigen carriers in Greece. Nosokomiaka Chronika.

[B6] Elefsiniotis IS, Glynou I, Pantazis KD, Fotos NV, Magaziotou I, Kada H (2005). Prevalence of chronic HBV infection among 13,581 women at reproductive age in Greece. A prospective single center study. J Clin Virol.

[B7] Papaevangelou G (1998). Hepatitis B immunization program: lessons learnt in Greece. Vaccine.

[B8] Centers for Disease Control and Prevention (2005). A comprehensive immunization strategy to eliminate transmission of hepatitis B virus infection in the United States: recommendations of the Advisory Committee on Immunization Practices (ACIP); Part 1: Immunization of Infants, Children, and Adolescents. MMWR.

[B9] Poland GA, Jacobson RM (2004). Prevention of Hepatitis B with the Hepatitis B Vaccine. N Engl J Med.

[B10] Mackenbach JP, Kunst AE, Cavelaars AE, Groenhof F, Geurts JJ (1997). Socioeconomic inequalities in morbidity and mortality in Western Europe. Lancet.

[B11] Sypsa V, Hadjipaschali E, Hatzakis A (2001). Prevalence, risk factors and evaluation of a screening strategy for chronic hepatitis C and B virus infections in healthy company employees. Eur J Epidemiol.

[B12] Dalekos GN, Zervou E, Karabini F, Tsianos EV (1995). Prevalence of viral markers among refugees from Southern Albania: increased incidence of infection with hepatitis A, B and D viruses. Eur J Gastroenterol Hepatol.

[B13] Dentinger CM, McMahon BJ, Butler JC, Dunaway CE, Zanis CL, Bulkow LR, Bruden DL, Nainan OV, Khristova ML, Hennessy TW, Parkinson AJ (2005). Persistence of antibody to hepatitis B and protection from disease among Alaska natives immunized at birth. Pediatr Infect Dis J.

[B14] Knoll A, Hartmann A, Hamoshi H, Weislmaier K, Jilg W (2006). Serological pattern "anti-HBc alone": characterization of 552 individuals and clinical significance. World JGastroenterol.

